# Tiotropium's cost-effectiveness for the treatment of COPD: a cost-utility analysis under real-world conditions

**DOI:** 10.1186/1471-2466-10-47

**Published:** 2010-09-15

**Authors:** Mattias Neyt, Stephan Devriese, Nancy Thiry, Ann Van den Bruel

**Affiliations:** 1 Belgian Health Care Knowledge Centre (KCE), Brussels, Belgium; 2 Academic Centre for Primary Care, KULeuven, Leuven, Belgium

## Abstract

**Background:**

Tiotropium is reimbursed since March 2004 in Belgium for the treatment of Chronic Obstructive Pulmonary Disease (COPD). Questions however remain on this product's value for money. The purpose of this study is to calculate tiotropium's cost-effectiveness under real-world conditions.

**Methods:**

Strengths of both observational and RCT data were combined in a model. A large longitudinal (2002-2006) observational dataset of regular tiotropium users (56 321 patients) was analysed to retrieve the baseline risk for exacerbations and exacerbation-related hospitalisations the year before the first delivery of tiotropium. The relative treatment effect from the UPLIFT (Understanding Potential Long-term Impacts on Function with Tiotropium) trial was then applied to this baseline risk to reflect the effect of tiotropium treatment and calculate the intervention's incremental cost-effectiveness ratio (ICER).

**Results:**

After 1000 Latin Hypercube simulations, the incremental benefit expressed as quality-adjusted life years (QALY) gained is on average 0.00048 (95% confidence interval (CI) 0.00009 - 0.00092). In combination with a substantial mean incremental cost of €373 per patient (95% CI 279 - 475), this results in an unfavourable average ICER of €1 244 023 (95% CI 328 571 - 4 712 704) per QALY gained. Results were most sensitive to the treatment effect on hospitalisations. Based on our large observational database, up to 89% of the patients were not hospitalised for COPD in the year before the first tiotropium delivery.

**Conclusions:**

The main cause for tiotropium's unfavourable cost-effectiveness ratio is a combination of a relative high price for tiotropium, a low number of hospitalisations without tiotropium treatment (on average 0.14/year) and a non-significant treatment effect (on average 0.94) with respect to avoiding exacerbation-related hospitalisations. From an economic point of view, a revision of reimbursement modalities (e.g. with a lower price) would be justified and would entail a more efficient use of resources.

## Background

Tiotropium (Spiriva^®^, Boehringer-Ingelheim) is a once-daily inhaled long-acting bronchodilator of the anticholinergic class, used for the maintenance treatment of Chronic Obstructive Pulmonary Disease (COPD). In Belgium, tiotropium is reimbursed since March 1, 2004. This decision was partly based on the claims that the budget impact for the National Institute for Health and Disability Insurance (NIHDI) would be offset by cost savings due to less hospital admissions and less use of antibiotics and oral corticosteroids. Being a Class I drug, i.e. a drug with an assumed therapeutic added value in comparison to existing alternatives, a revision of the reimbursement decision was required within 36 months. The conclusions of this revision indicated that there was no benefit of using tiotropium in comparison to long-acting β2-agonists, that the price of tiotropium was higher than these alternatives, and that there was no evidence of the introduction of tiotropium decreasing treatment costs related to other drugs[1]. Nevertheless, reimbursement modalities remained unchanged after this revision. Since then, results of the large UPLIFT (Understanding Potential Long-term Impacts on Function with Tiotropium) trial have been published, which are taken into account in this economic evaluation.

In Belgium, with a total population of about 10.6 million inhabitants, NIHDI reimbursements for tiotropium were more than €26 million in 2007 for 86 000 patients with at least one delivery of tiotropium. One month of tiotropium costs €51.75 per patient, whereas e.g. salmeterol, i.e. another long-acting bronchodilator, costs €31.24 per month.

The goal of this study, part of a full Health Technology Assessment (HTA) report performed by the independent Belgian Health Care Knowledge Centre,[2] is to calculate the cost-effectiveness of tiotropium under real-world conditions.

## Methods

The basic idea is to use the strengths of both observational and RCT data. RCTs are the ideal method for measuring treatment effects. Randomization reduces biases by making treatment and control groups "equal with respect to all features," except the treatment assignment[3]. Nevertheless, the population in trials often does not reflect a real-world population. An approach to measure the cost-effectiveness for this population is to apply the relative treatment effect found in RCTs to the baseline risk for an event calculated from observational data. As such, the incremental cost-effectiveness ratio (ICER), which is driven by absolute benefit, will be lower (i.e. more cost-effective) in subgroups with a higher baseline risk of a certain event (such as exacerbations and exacerbation-related hospitalisations), and vice versa[4]. This is under the assumption that the relative treatment effect is independent from the baseline risk. In the absence of published evidence on a difference in relative treatment effect between subgroups, this may be seen as a realistic assumption.

The calculation contains three steps: a) what is the cost and frequency of events for COPD patients not taking tiotropium (based on observational data); b) how many events would have occurred if these patients would have been treated with tiotropium (applying the relative treatment effect derived from the RCT on these observational data); and c) what is the ICER comparing the incremental costs and benefits with and without tiotropium treatment?

### Decision model

The analysis is performed from the perspective of the health care payer, including both costs paid by the standard health insurance and patient co-payment contributions. A review on the efficacy/effectiveness of tiotropium showed no impact of tiotropium on survival[2]. However, avoiding exacerbations and exacerbation-related hospitalisation may influence quality of life (QoL). Therefore, a cost-utility analysis is performed. There is no evidence that the long-term course of the disease is altered by using tiotropium in comparison with its relevant comparators, such as salmeterol. Therefore, for this chronic disease, a short-term time horizon of one year is applied. Moreover, one year appears to be long enough to capture a significant number of important clinical endpoints such as exacerbations and exacerbation-related hospitalisations, and to capture seasonal variations. Due to the short time horizon, no discount rate was applied.

**Figure 1:**
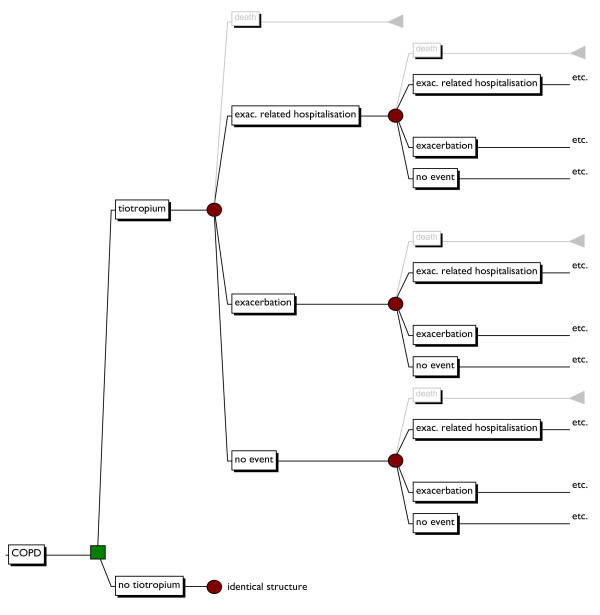
**Decision model of tiotropium for the treatment of COPD**. (Additional file). exac.: exacerbation. Items shown in grey are not taken into account.

The structure of the model is shown in figure 1. The disease course of COPD is more complex than this model shows. However, to calculate an intervention's cost-effectiveness, only the difference in (i.e. incremental) costs and effects are of importance. For example, since there is no univocal proven impact on mortality, this event is not taken into account.

### Tiotropium study database

Data on health care use, patient characteristics, and pathology information of tiotropium users was drawn from two existing data sources: the IMA (Common Sickness Funds Agency) claims database and the MCD-HBD (Minimal Clinical Data - Hospital Billing Data) pathology and claims database of in hospital stays from the TCT (Technical Cell). The IMA claims database contains the detailed registration of all ambulatory and in hospital health care use reimbursed by the Belgian compulsory health insurance. The TCT MCD-HBD database contains the detailed registration of both reimbursements and ICD9-CM codes of all Belgian in hospital health care use. IMA claims and patient characteristics data from 2002 to 2006 for patients for which the NIHDI code 00470448 (Spiriva, tiotropium) and corresponding CNK (pharmaceutical product code) codes indicated a tiotropium delivery between March 1, 2004 and December 31, 2006, was requested. For the selected patients, all MCD-HBD hospital stay data from 2002 to 2005 were obtained. As such, observational data one year before and after the first delivery of tiotropium was gathered. A detailed description of these data sources and the technicalities of the construction of the tiotropium study database can be found in the appendix of the full HTA[2].

In this database, the first delivery of tiotropium to a patient can fall between March 1, 2004 and December 31, 2005. As a result, the year before the first delivery can fall between March 1, 2003 and December 31, 2005. The year after the first delivery can start for some patients on March 1, 2004 up to December 31, 2005 and end somewhere between March 1, 2005 and December 31, 2006. Information on baseline risks for exacerbations and exacerbation-related hospitalisations without tiotropium is based on the year before the first tiotropium delivery. Details on the costs for tiotropium were based on the first year after the first delivery. Information on costs for exacerbation-related hospitalisations was based on the complete database.

An authorization for the processing and analyses of this database was obtained from the Belgian Privacy Commission[5].

#### Population

The initial database of patients with at least one registration of tiotropium included 102 796 patients. From this database, an algorithm was used to select patients that used tiotropium on a more regular basis (more than one delivery on separate days, total number of daily defined dosis (DDDs) ≥ 90 in one year, reimbursement for tiotropium ≥ €120 in one year, DDD ratio ≥ 90/365, at least one year of data before and after the first delivery for tiotropium). 56 321 patients (54.79%) fulfilled all criteria. The average age is 68.05 years and 66.39% are male. These patients should all have been diagnosed with COPD since in Belgium reimbursement of tiotropium is restricted to this indication.

#### Medication

The costs, including both the NIHDI reimbursement and patient co-payment, were on average €427.71 for tiotropium (table 1). The observational database showed there is no clear indication for a decrease in the use of other medication when patients started taking tiotropium. Therefore, these costs were left unchanged in the base case scenario.

**Table 1:** Input parameters for the economic evaluation

Input variable		Base-case value	Range (95% CI)		Source
**Costs (€)**					
medication					
*tiotropium*	real-world^a^	427.71	426.25	429.17	Belgian database
	theoretical	621.00	/	/	BCFI
*salmeterol*	real-world^b^	225.46	223.00	227.92	Belgian database
exacerbation	theoretical	52.72	41.34	64.48	result 1000 simulations
exac.-rel. hosp.		5617	5555	5680	Belgian database
**Utilities**					
exac.-rel. hosp.	QALYgained^c^	0.013	0.004	0.019	result 1000 simulations
*at admission*		-0.077	-0.58	0.79	O'Reilly et al.[9]
*at discharge*		0.58	-0.16	0.98	O'Reilly et al.[9]
*LOS (days)*		14.18	14.02	14.34	Belgian database
exacerbation	QALYgained^c^	0.003	0.001	0.005	result 1000 simulations

**Events (average per patient)**					
exacerbations		0.800	0.775	0.826	Belgian database and Roede et al.[6]
exac.-rel. hosp.		0.141	0.137	0.144	Belgian database

**Efficacy/effectiveness**					
exacerbation	relative risk^d^	0.86	0.81	0.91	Tashkin et al.[8]
exac.-rel. hosp.	relative risk^d^	0.94	0.82	1.07	Tashkin et al.[8]

BCFI: Belgian Centre for Pharmacotherapeutic Information; LOS: length of stay; NIHDI: National Institute for Health and Disability Insurance; QALY: quality-adjusted life year

a: based on the first year of tiotropium use

b: based on the year before the first delivery of tiotropium

c: average QALY gained per event avoided

d: using the published 95%CI to define our distribution results in the following mean values in the model: 85.85% instead of 86% and 93.67% instead of 94%.

#### Exacerbations

Since no ambulatory clinical data were available, exacerbations are defined on the basis of health care resources use. The proxy used was the delivery of both oral corticosteroids and antibiotics within 7 days. As such, the number of exacerbations was underestimated since in practice not all exacerbations are treated this way. A Dutch study found that steroids combined with an antibiotic were prescribed in 23% of exacerbations[6]. This factor was taken into account in our model resulting in 0.8 exacerbations on average per patient during one year, which was in line with the yearly exacerbation frequency in the UPLIFT trial (0.85 in the placebo group).

The cost per exacerbation was defined theoretically. The dosis of bronchodilators may be increased and oral steroids (7-10 days 30-40 mg prednisolone) and antibiotics (3-7 days in case of certain symptoms) may be prescribed. For our calculation, the costs for oral steroids and antibiotics take into account a uniform distribution on the number of days, i.e. 7-10 days and 3-7 days for both drugs, respectively. The price for prednisolone was €31.5 for 20 units of 32 mg, i.e. €1.58 per day. With respect to antibiotics, a daily cost of €1.13 was taken into account (amoxicilline (Docamoxi - 500 mg) taken 3 times a day (1500 mg/day) costs €6.02 for 16 units). One to two visits (uniform distribution) to the general practitioner (€22.46 per visit) were also added. As a result, the price for an exacerbation not requiring hospitalisation was on average €52.72 (Table 1).

#### Exacerbation-related hospitalisations

All hospitalisations except for one day admissions for the selected patients were at our disposal. To calculate the number of COPD related hospitalisations, only those stays with ICD-9-CM codes (and subcodes) 491 (chronic bronchitis), 492 (emphysema) and 496 (COPD, not otherwise specified) as the primary diagnosis in any hospital ward during the stay were retained.

This resulted in a rate of 0.14 (SD 0.46) hospitalisations per year (Table 1), comparable to the 0.16 rate in the placebo arm of the UPLIFT trial.

To calculate the cost of a COPD hospitalisation, an additional, more stringent, selection criterion was used: the hospital stay must be spend either in a single hospital ward, or in two hospital wards of which one was the intensive care unit. This avoided an impact on hospitalisation cost of interventions unrelated to COPD in the same stay in other hospital wards. The cost was calculated as the sum of all expenditures available in the MFG data for the selected stay. In Belgium, hospital per diem costs are covered by 2 distinct systems of public health funding. A major part is covered through fixed monthly hospital payments but these are not registered in the MFG data. Additional remuneration consists of a lump sum billed per admission and a lump sum billed per day of hospital stay, both included in the MFG data. These lump sums were replaced by the 100% hospital per diem costs calculated as the actual per diem prices available per hospital, per year, per semester and per type of stay (published by NIHDI[7]) multiplied by the number of invoiced days for the stay. The average length of stay was 14.18 (SD 16.01) days and the COPD-related hospitalisation cost was €5617 (SD 6023).

### Treatment effect

In October 2008, results of the UPLIFT trial have been published. In this randomized, double-blind trial, 4 years of tiotropium treatment was compared with placebo in patients with COPD who were permitted to use all respiratory medications except inhaled anticholinergic drugs. The patients were at least 40 years of age, with a post-bronchodilator FEV1 of 70% or less than the predicted value and a ratio of FEV1/FVC of 70% or less. In this very large trial, 2987 and 3006 patients were randomly assigned to the tiotropium and placebo group, respectively[8]. The hazard ratio for COPD exacerbations was 0.86 (95% CI: 0.81 - 0.91; p-value <0.001). For exacerbations leading to hospitalisations this was 0.94 (95% CI: 0.82 - 1.07; p-value = 0.34).

In the tiotropium study database, it was observed that there was no reduction in the use of other medication after the first delivery of tiotropium. For example, in both the year before and after the first tiotropium delivery, 80% of patients purchased at least one package of long-acting beta-agonists, salmeterol or formoterol, either in individual formulation or in combination with an inhaled corticosteroid. In other words, in the real world, tiotropium does not always replace the other long-acting bronchodilators and the comparator is a combination of other drugs. Similarly, in the UPLIFT trial, other drugs were taken and a long-acting inhaled β2-agonist was used in 60.1% of both treatment arms and tiotropium was added in one treatment arm[8]. Therefore, both because of its size and because it seems to correspond to what happens in reality, the treatment effects and the surrounding uncertainty from the UPLIFT trial are taken into account to calculate the real-world cost-effectiveness of tiotropium.

### Utilities

Tiotropium may avoid exacerbations and exacerbation-related hospitalisations. Next to changes in costs, this also entails changes in utilities. Therefore, for both exacerbations and exacerbation-related hospitalisations QoL data were gathered. One article explicitly stated general QoL outcomes associated with COPD exacerbation managed in a UK-based hospital, using the EQ-5 D questionnaire, including 149 patients representing 222 admissions to hospital[9]. At admission, the mean utility (-0.077, SD: 0.397) indicated great impairment, with 61% of patients having a negative utility value representing a health state equivalent to 'worse than death'. Great improvements were reported during admission and at discharge, where the mean utility value was increased to 0.576 (SD: 0.317). This deterioration and improvement in QoL was included in our model for exacerbation-related hospitalisations. A correlation of 0.9 was implemented between the value at admission and discharge to avoid increases of more than 1.1584 (i.e. the maximum difference according to the Flemish EQ-5 D utility scale) in our probabilistic model.

No studies were identified reporting changes in QoL during exacerbations measured with a generic QoL instrument. Several studies made assumptions on the QoL deterioration. A reduction of 15% in QoL for one month after a (moderate) exacerbation and 50% or 70% for a severe exacerbation (requiring hospitalisation) was assumed,[10-13] or a relative ratio of 3.33 or 4.67. In our model, the average relative ratio of 4 was applied on the reduction in QoL for an exacerbation-related hospitalisation, with a minimum of 3.33 and a maximum of 4.67 (uniform distribution).

### Scenario analysis

Next to the base case, two scenarios are elaborated. A first scenario incorporated the theoretical yearly cost for tiotropium (€621) and excluded the observed expenditures for salmeterol (on average €225.46), reflecting 100% tiotropium compliant users that did not take salmeterol anymore. In a second scenario, the ICER was calculated depending on the initial number (i.e. without tiotropium) of exacerbations and hospitalisations. Details and results of other scenario analyses can be found in the full HTA report[2].

### Probabilistic modelling

To capture parameter uncertainty, input variables in our model are probabilistic values. The choice of distribution depends on the characteristics of the input variables. Due to the central limit theorem, cost parameters retrieved from the large tiotropium study database were sampled from a normal distribution with the appropriate confidence interval around the mean (table 1). Uniform distributions were included were we had no idea of the distribution (e.g. in our theoretically calculated exacerbation cost). The beta distribution, which is constrained on the interval 0-1, is the ideal distribution for QoL values. Since the study of O'Reilly et al[9]. provided QoL data during an exacerbation-related hospitalisation with negative values, an adjusted beta distribution with the reported minimum value of -0.59 and a maximum of 1 was applied. Finally, the relative risk parameters are modelled using the lognormal distribution, reflecting the published mean values and 95% CI. 1000 Latin hypercube simulations were generated in MicroSoft Excel using the @Risk (Palisade Corporation) add-in program. Following the Belgian pharmacoeconomic guidelines, the central estimate of the ICER results directly from the probabilistic sensitivity analysis as the mean of the simulated ICERs. This is not necessary equal or close to the ratio of the mean incremental cost and mean incremental effect, which is the deterministic version of the ICER. A deterministic ICER is presented if the Latin Hypercube simulations fall in different quadrants of the cost-effectiveness plane[14].

## Results

### Base case scenario

**Figure 2:**
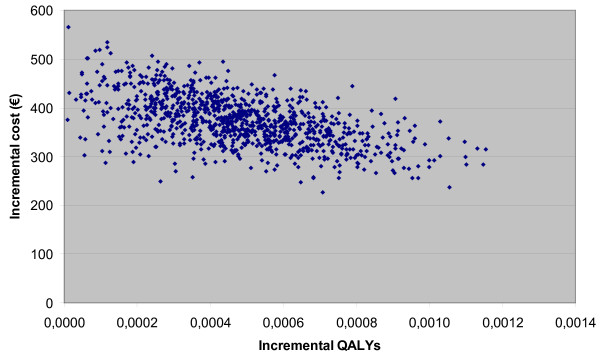
**The cost-effectiveness plane**. (Additional file). QALY: quality-adjusted life year.

**Figure 3:**
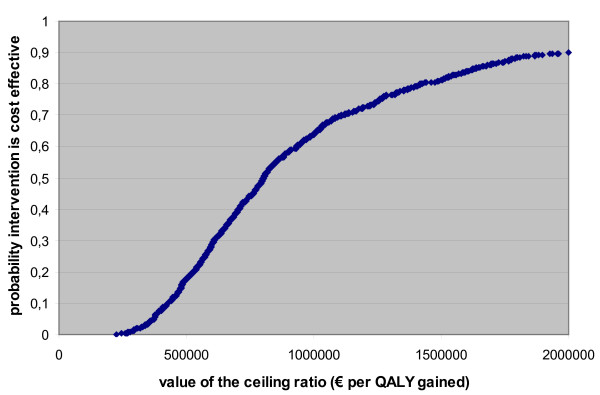
**The cost-effectiveness acceptability curve**. (Additional file). QALY: quality-adjusted life year.

The mean incremental cost is €373 per patient (95% CI 279 - 475). This incremental cost is composed of the following three elements: incremental cost medication: €427.71 (95% CI 426.25 - 429.17), incremental cost related to hospitalisations: -€48.26 (95% CI -143.11 - 55.61), and the incremental cost related to exacerbations: -5.95 (95% CI -8.76 - -3.56). The UPLIFT non-significant treatment effect on hospitalisations and the significant treatment effect on exacerbations are reflected in these confidence intervals. The incremental benefit expressed as quality-adjusted life years (QALY) gained are on average 0.00048 (95% CI 0.00009 - 0.00092). This is relatively low due to the combination of the following factors: a) a low number of hospitalisations without tiotropium treatment (on average 0.14/year), b) a non-significant treatment effect (on average 0.94) with respect to avoiding exacerbation-related hospitalisations, and c) the relatively short duration that this event influences QoL. In combination with the non-negligible incremental costs, this results in an ICER of €1 244 023 (95% CI 328 571 - 4 712 704) per QALY gained. Results are presented on the cost-effectiveness plane (Figure 2) and cost-effectiveness acceptability curve (Figure 3). The latter shows that the treatment can only be considered cost effective at relatively very high willingness-to-pay values. The results are most sensitive to the treatment effect on hospitalisations (correlation coefficient 0.671).

### Scenario analyses

In the scenario with 100% compliant tiotropium users which stopped taking salmeterol, the incremental cost decreases to €341 (95% CI 246 - 445). In combination with the same incremental benefit, this results in an ICER of €1 142 478 (95% CI 295 004 - 4 396 273) per QALY gained.

Table 2 presents the cost-effectiveness of tiotropium depending on the initial number of exacerbations and/or hospitalisations. It shows that the number of hospitalisations in particular determines tiotropium's cost-effectiveness. Even with 10 exacerbations without tiotropium, the average ICER is above €100 000 per QALY gained if the patient has no hospitalisation without tiotropium. Based on our large observational database, 89% of the patients (i.e. 50 156 patients) was not hospitalised for COPD in the year before the first tiotropium delivery. For patients who would not have been hospitalised without tiotropium, there is not much to gain by treating them with tiotropium. Only 2.2% had two or more COPD-related hospitalisations prior to tiotropium treatment.

**Table 2:** IC, IE and ICERs depending on the initial number of hospitalisations and exacerbations

**prior n° of exac**.	**prior number (n°) of hospitalisations**															
	**0**						**1**					**2**				
			
	**IC**		**IE**		**ICER**		**IC**		**IE**		ICER*	**IC**		**IE**		ICER*
		
**0**	428		0.0000		dominated		84		0.0008		*107666*	-259		0.0016		*cost saving*
	426	429	0.0000	0.0000			-581	819	-0.0009	0.0027		-1589	1211	-0.0017	0.0054	
		
**1**	420		0.0005		1265951		77		0.0012		*62094*	-266		0.0020		*cost saving*
	416	424	0.0001	0.0008	497057	4154369	-590	812	-0.0004	0.0033		-1599	1204	-0.0013	0.0061	
		
**2**	413		0.0009		622357		70		0.0017		*41019*	-274		0.0025		*cost saving*
	406	419	0.0002	0.0017	242740	2049176	-599	805	0.0000	0.0039		-1608	1196	-0.0008	0.0066	
		
**3**	405		0.0014		407826		62		0.0022		*28873*	-281		0.0029		*cost saving*
	395	415	0.0003	0.0025	158255	1338516	-608	797	0.0002	0.0046		-1617	1189	-0.0003	0.0072	
		
**4**	398		0.0018		300561		55		0.0026		*20974*	-289		0.0034		*cost saving*
	384	410	0.0004	0.0034	116258	983186	-618	790	0.0003	0.0053		-1626	1182	0.0000	0.0077	
		
**5**	391		0.0023		236201		47		0.0031		*15425*	-296		0.0038		*cost saving*
	374	406	0.0005	0.0042	90683	769988	-627	780	0.0005	0.0061		-1635	1175	0.0003	0.0085	
		
**6**	383		0.0027		193295		40		0.0035		*11314*	-303		0.0043		*cost saving*
	363	401	0.0006	0.0050	74097	627857	-634	770	0.0007	0.0068		-1645	1167	0.0004	0.0092	
		
**7**	376		0.0032		162648		32		0.0040		*8146*	-311		0.0048		*cost saving*
	352	397	0.0007	0.0059	62023	529076	-641	760	0.0008	0.0076		-1654	1160	0.0006	0.0099	
		
**8**	368		0.0036		139662		25		0.0044		*5630*	-318		0.0052		*cost saving*
	341	392	0.0008	0.0067	52879	456718	-650	750	0.0010	0.0084		-1663	1153	0.0007	0.0106	
		
**9**	361		0.0041		121784		18		0.0049		*3583*	-326		0.0057		*cost saving*
	330	388	0.0009	0.0075	45609	400440	-659	740	0.0011	0.0092		-1672	1143	0.0008	0.0114	
		
**10**	353		0.0046		107482		10		0.0053		*1885*	-333		0.0061		*cost saving*
	319	383	0.0010	0.0084	39741	355417	-667	730	0.0012	0.0100		-1681	1133	0.0010	0.0122	

Exac. = exacerbations

IC: incremental cost (expressed in euro); IE: incremental effect (expressed in quality-adjusted life years (QALY) gained); ICER: incremental cost-effectiveness ratio (expressed in cost per QALY gained). In every cell, the mean value and the lower (2.5%) and upper (97.5%) limits of the interval are mentioned.

* the ICERs of the probabilistic model are not mentioned since simulation results are spread over several quadrants of the cost-effectiveness plane. As an alternative (in italics), the ICER calculated by dividing the mean incremental cost by the mean incremental benefit is presented.

## Discussion

A disadvantage of our database is that no parameter was present indicating the COPD stage. As a result, no subgroup analyses could be made according to these stages. In our approach, the population was initially analysed as a whole (i.e. all patients fulfilling our inclusion criteria). In subgroup analyses, the population was divided depending on the number of hospitalisations related to exacerbations (i.e. one, two, three or more exacerbation-related hospitalisations) in combination with the number of exacerbations. These subgroup analyses showed that, in the first place, for a selected group of patients with a relative high base risk on events, the results were more cost effective. On the other hand, it also showed that this is only a minority of the population taking tiotropium on a regular basis. The largest group of patients did not experience any exacerbation or hospitalisation. For this group of patients no events can be avoided because they do not occur without taking tiotropium, no QoL can be gained, no costs will be saved, and tiotropium would only induce higher costs.

As mentioned by Drummond, "The assumption of constant relative effects being applied to subgroup-specific baseline event rates is common in cost-effectiveness model."[4] A general criticisms is that it may be questionable whether the relative treatment effect is independent from the baseline risk since subgroup analysis often show the opposite. On the other hand, caution is needed when drawing conclusions from subgroup findings[15]. It is generally recognized that subgroup analyses can produce spurious results[16]. Some authors even argued that "the overall 'average' result of a randomised clinical trial is usually a more reliable estimate of treatment effect in the various subgroups examined than are the observed effects in individual subgroups"[17]. Furthermore, even if the relative treatment effect would be higher in a specific subgroup, than still the absolute gain (which determines the cost-effectiveness) would be limited due to the low baseline risk for events without tiotropium treatment occurring in our real-world population.

Our health technology assessment included a systematic review of the economic literature. Details of the search strategy are available in the full HTA report[2]. Eight full economic evaluations of tiotropium were identified, all published after 2004. Analyses were performed for the Netherlands,[12,18] Canada,[12,19] Switzerland,[20] Greece,[11] Spain,[13] and the US[21,22]. Two studies were trial-based economic evaluations[18,19]. The US studies were deterministic[21] or probabilistic[22] simulations using aggregated data depicting a typical COPD patient. The remaining four studies were Markov model-based economic evaluations, all built on an initial model developed by Oostenbrink et al[12]. All studies limited their time horizon to one year, with exception of one model-based evaluation using a 5-year time horizon[13]. Surrogate outcome measures of the drugs' clinical action were used in cost-effectiveness analyses: exacerbation-free months gained[13] or exacerbations avoided[11,12,18-20,22]. These outcomes are rather difficult to interpret by decision makers, e.g. being not comparable with results of studies in other indications.

Four studies compared tiotropium with salmeterol[11-13,20]. The mean incremental outcomes in these analyses (whether QALYs gained, exacerbations avoided or exacerbation-free months gained) were associated with large 95% confidence intervals crossing zero[11-13]. One study found tiotropium to be dominant,[20] however, this result was determined deterministically not including uncertainty around the treatment effect. Two studies found that there was almost neutrality between the two alternatives in terms of incremental costs and QALYs, as the dots simulated were almost evenly scattered around the four quadrants of the cost-effectiveness plane[11,12]. In the cost-utility analysis with a 5-year-time horizon, approximately 80% of the simulations were in the right quadrants of the cost-effectiveness plane. The gain in QALYs remained however small (0.14) and non-significant (95% CI: -0.16-0.49). Based on the ratio of the mean incremental costs to the mean incremental QALYs, they found a cost-effectiveness ratio of €4118 per QALY gained[13]. We discuss some differences between these analyses.

One of the strengths of our evaluation is that it considers patients that use tiotropium in their everyday life. In contrast, populations considered in previous analyses were more selective. All previous analyses, with the exception of one,[22] excluded patients with mild COPD. Nevertheless, these patients are also included in the Belgian reimbursement modalities for tiotropium. This can be very determining for the results of an economic evaluation. The base risk for certain events may be different in comparison to events in trials including a selective population. It is the combination of this base risk and the relative treatment effect that determines the absolute reduction in events, which determines the intervention's cost effectiveness.

Next, there are differences in applied treatment effects. Studies comparing tiotropium with salmeterol relied on the trial results published by Brusasco et al[23]. At that time, results of the UPLIFT trial were not published yet. These trials are not designed to detect a difference in exacerbation-related hospitalisations. It is therefore important to take into account the uncertainty around these estimations. The mean treatment effect in the UPLIFT trial is 0.86 (95% CI 0.81 - 0.91, p-value <0.001) for exacerbations and 0.94 (95% CI 0.82 - 1.07, p-value = 0.34) for exacerbations leading to hospitalisations. The mean treatment effect based on the Brusasco trial is similar for exacerbations, i.e. 0.87 (p-value = 0.22), but much more favourable for hospitalisations, being 0.59 (non-significant but no p-value published). Both estimates are surrounded by much uncertainty. Furthermore, it should be remarked that a critical review of the literature identified the presence of publication bias[2]. A second trial[24] also reported non-significant p-values for the difference in exacerbation and hospitalisation frequencies. However, no exact results (even no mean values) were provided and results could therefore not be included in a meta-analysis.

There were also large cost differences between the identified studies, both with respect to the cost of medication, treatment of exacerbations and cost of hospitalisations. For example, a non-severe exacerbation costs between €47 in Canada[12] against nearly €1000 in Greece[11]. Similarly, a severe exacerbation costs €2660 in Spain[13] against €4400 in Greece,[11] and even over €4600 in the US[22]. In our study the cost for an exacerbation not requiring hospitalisation was on average €52.72 and the COPD-related hospitalisation cost was €5617. In general, the higher these event-related costs (which are context specific), the more favourable study results are for tiotropium. Of course, the impact depends on the combination of the baseline risk for an event, the treatment effect and its costs.

Most economic evaluations used a 1-year timeframe and assumed tiotropium had no influence on mortality. Only one analysis took a 5-year time horizon reflecting the progressive nature of COPD[13]. The authors note that no differences between treatments (tiotropium, salmeterol or ipratropium) in terms of mortality risk were assumed. Also the costs per disease severity state and per severe or non-severe exacerbation were mentioned to be equal across treatment groups. In their discussion, the authors repeat that the difference between the three treatment groups in terms of QALYs were small, which was expected because treatments do not directly affect survival[13]. However, in this analysis, mortality is influenced when comparing the three treatment arms due to different transition probabilities between the disease states for the alternative treatments. In other words, the chance of dying when a patient is in a certain disease state is equal over the three groups with the highest probability in the very severe disease state. Nonetheless, the transition probability to go to the more severe disease states was modelled to be higher for salmeterol and ipratropium. As a result, this model implicated an implicit effect on mortality. Replicating the model with the same probabilities as in the published paper, the 5-year mortality was about 15% with tiotropium and about 20% with salmeterol and ipratropium. Absence of evidence is not evidence of absence. However, based on currently available evidence, indirectly modelling a relatively large impact on mortality can be considered as a rather optimistic scenario.

Impact on events which may influence QoL is one of the main drivers of results. Unfortunately, accurate utility (or disutility) scores for events on which tiotropium may have an impact, i.e. exacerbations and exacerbation-related hospitalizations, are still lacking. We included data reflecting on the impact on QoL during hospitalisation. It was previously remarked that low values reported during the stay in hospital partly constitute an effect of the exacerbation and partly of the hospitalisation. The improving figures after returning home are probably due to a change in both medical status and environment[25]. Especially the fact of being hospitalised may influence QoL. However, we currently do not know whether further improvements are observed afterwards in real life. Three studies including EQ-5 D health states estimated that utility values would reduce by 15%[26] in case of non-severe exacerbation, and by 50%[27] in case of severe exacerbation[11-13]. Nevertheless, the referenced papers to support these figures do not mention such data. It would be interesting if researchers try to measure the impact of these events on QoL (in complement to measuring QoL on pre-specified points in time, since these events may occur in between). This would enable a more evidence-based calculation of impact on QALYs[28].

## Conclusion

This is the first economic evaluation that tries to calculate tiotropium's cost-effectiveness for a population treated in every-day practice. This is done by combining three elements in a model: a) the number of events without tiotropium (the baseline risk); b) tiotropium's treatment effect on these events; and c) the changes in QoL by avoiding an event. For the latter, assumptions had to be made due to a lack of evidence. Further research on the impact of exacerbations on a patient's QoL (measured with a generic instrument useful for economic evaluations) would be interesting. For the other two elements, the published treatment effects from the UPLIFT trial were applied on the baseline risk for exacerbations and exacerbation-related hospitalisations observed in a large Belgian database. The cost-effectiveness of tiotropium was unfavourable due to the low average of hospitalisations with standard care but without tiotropium treatment (almost 90% did not experience an exacerbation-related hospitalisation). As a result, treating this part of the population with tiotropium mainly results in extra costs without benefits.

From a medical point of view, tiotropium earns its place in the treatment of COPD. As shown in a systematic review of the literature, tiotropium was found to have statistically significant effects compared to placebo and ipratropium, but not compared to salmeterol[2]. Furthermore, the GOLD (Global Initiative for Chronic Obstructive Lung Disease) guideline mentions that regular treatment with long-acting bronchodilators is more effective and convenient than treatment with short-acting bronchodilators. The choice between β2-agonists, anticholinergics, methylxanthines, or combination therapy depends on availability and individual response in terms of symptom relief and side effects. However, there is insufficient evidence to favour one long-acting bronchodilator over another[29].

To date, tiotropium has not clearly demonstrated an important effect on QALYs nor generated large cost savings by avoiding COPD related events in comparison to standard treatment (real world situation) or in comparison with salmeterol. Since tiotropium does not perform worse but also not much better than its comparator on patient-relevant outcomes, the higher price of tiotropium can be questioned from a payer's perspective. Price negotiations could be started in a revision procedure of the reimbursement modalities. If tiotropium's price would for example be reduced to €31.24 for 30 units, i.e. the price in Belgium for the long-acting bronchodilator salmeterol, than the budget impact for both the NIHDI and the patient's co-payment, based on 2007 data, would decrease to about €16 million, or a cost saving of more than €10 million a year. These resources could then be invested more efficiently. With current pressure on budgets, these efficiency considerations should not be neglected in resource allocation decisions.

## Competing interests

The study was not externally funded. The authors declare that they have no competing interests.

## Authors' contributions

All authors participated in the concept and design of the study. STD participated in data collection, and MAN and STD in data analysis. MAN performed the economic evaluation. All authors read and approved the final manuscript.

## Pre-publication history

The pre-publication history for this paper can be accessed here:

http://www.biomedcentral.com/1471-2466/10/47/prepub
